# Factors That Influence Compliance to Long-Term Remote Ischemic Conditioning Treatment in Patients With Ischemic Stroke

**DOI:** 10.3389/fneur.2021.711665

**Published:** 2021-08-30

**Authors:** Jie Zhao, Kaiting Fan, Wenbo Zhao, Hui Yao, Jiayue Ma, Hong Chang

**Affiliations:** Department of Neurology, Xuanwu Hospital, Capital Medical University, National Clinical Research Center for Geriatric Disease, Beijing, China

**Keywords:** patient compliance, remote ischemic conditioning, influencing factor, ischemic stroke, secondary prevention

## Abstract

**Objectives:** To investigate the treatment compliance of patients with ischemic stroke to remote ischemic conditioning (RIC) and to determine the factors that influence compliance.

**Methods:** We conducted a retrospective study of patients with ischemic stroke who were treated with RIC. Treatment compliance was determined and analyzed in patients who had received 1 year of RIC training. Factors that influenced patient compliance were also determined using univariate and multivariate regression analyses.

**Results:** Between March 2017 and February 2018, 91 patients were recruited into this study. The mean (±SD) age was 57.98 ± 10.76 years, and 78 (85.7%) patients were male. The baseline Kolcaba comfort scale of patients with good compliance scores were higher than those with poor compliance. The scores of the four dimensions in the scale and the total score are as follows: physiological dimensions, 15.0 (12.0,17.0) vs 17.0 (13.0,19.0); psychological dimensions, 30.0 (25.0,34.0) vs 31.0 (27.0,35.0); sociological dimensions, 20.0 (18.0,24.0) vs 21.0 (18.0,23.0); environmental dimensions, 19.0 (12.0,24.0) vs 20.0 (17.0,22.0); and total points, 82.0 (69.0,94.0) vs 91.0 (78.0,98.0). the differences between the groups were significant (*p* < 0.05), except for the sociological dimensions. A history of hypertension, number of follow-ups, and the physiological, psychological, and environmental dimensions of the comfort scale were related to patient compliance, out of which the number of follow-ups (Adjusted OR = 2.498, 95% confidence interval (CI) 1.257–4.964) and the physiological discomfort (Adjusted OR = 1.128, 95% CI 1.029–1.236) independently influenced compliance (*p* < 0.05).

**Conclusion:** In patients with ischemic cerebrovascular disease who were treated with RIC, the number of follow-up visits and physiological discomfort associated with RIC treatment independently influenced patient compliance. Further studies are needed to investigate the RIC protocols and their corresponding nursing models.

## Introduction

Stroke is the second leading cause of death worldwide and the leading cause of death in countries such as China (1). Furthermore, ischemic stroke is the main subtype of stroke and accounts for 60–80% of all strokes (2). Treatment strategies for acute ischemic stroke (AIS) and its secondary prevention have been advanced significantly in the past decades; however, the prognosis of patients with AIS remains far from satisfactory. Therefore, effective adjuvant therapies for the treatment and secondary prevention of ischemic stroke are needed.

Remote ischemic conditioning (RIC), a non-invasive and easy-to-use method of physical therapy, which encompasses several cycles of ischemia/reperfusion training of an organ (e.g., limbs), confers protection to remote vital organs (3) and has been found to reduce recurrent strokes in patients with symptomatic intracranial artery atherosclerosis (4). It has also been found to improve cognitive function in patients with cerebral small vessel disease (5). Although its mechanism is not fully understood, studies have found that RIC could exert its protective effects immediately after the procedure, and this could last for 3–4 days with a 12-h unprotected interval (6). Therefore, RIC has been recommended for days or months, and previous clinical studies have reported protocols of RIC that range from once daily for 1 week to twice daily for 2 weeks, 6 months, and 1 year (7–9). For patients undergoing RIC for several months or years, compliance to RIC treatment is important to guarantee its protective effects. However, the compliance of patients with AIS to RIC treatment in real-world clinical practice and those factors that influence compliance remain unclear.

In this study, we aimed to investigate the compliance of patients with AIS who underwent repeated RIC for 1 year to RIC treatment and to determine the factors that influence compliance to RIC treatment.

## Methods

### Study Design and Participants

This study was based on a prospective randomized controlled trial that investigated RIC in patients with AIS (registered on www.chictr.org.cn/index.aspx with ChiCTR1800014403). Patients with ischemic stroke who participated in the randomized trial between March 2017 and February 2018 and who received RIC were recruited into this study. The inclusion criteria were as follows: (1) age ≥18 years; (2) NIH Stroke Scale (NIHSS) score of 0–7; (3) patients who were expected to benefit from non-surgical treatment program; (4) preliminary ultrasound examination excluding extracranial and subclavian artery stenosis with clear intracranial artery stenosis; and (5) patients with stroke that occurred within 30 days. The exclusion criteria were as follows: (1) life expectancy of <12 months; (2) acute bleeding diathesis (platelet count <100,000/mm^3^), heparin received within 48 h resulting in abnormally elevated activated partial thromboplastin time (aPTT) levels above the upper limit of normal, current use of anticoagulant with international normalized ratio (INR) >1.7 or prothrombin time >15 s, current use of direct thrombin inhibitors or direct factor Xa inhibitors with laboratory tests having increased sensitivity (e.g., aPTT, INR, platelet count, ecarin clotting time, thrombin time, or appropriate factor Xa activity assays); (3) skin diseases or fractures of the upper limbs; (4) pregnancy; (5) incomplete or missing data; and (6) long-term plans to stay abroad during the research period.

The study protocol was approved by the Ethics Committee of Xuanwu Hospital, Capital Medical University (2018009). All participants or their legally authorized representatives provided written informed consent.

### Interventions

All patients with AIS were treated according to physician's best judgment: aspirin alone (100–300 mg daily), clopidogrel alone (75 mg daily), or a combination of aspirin and clopidogrel, and antidiabetic or antihypertension when necessary. In addition, all patients received bilateral upper limb RIC, which was performed by an electric auto-control device (Patent No. ZL200820123637.X, China) (4) comprising five cycles of inflation and deflation for 5 min alternately, twice daily for 1 year, with an inflating pressure of 200 mmHg. RIC procedures were performed with the help of an assistant nurse in the hospital or their caregivers after they had been discharged from the hospital.

### Data Collection

Data collected included demographic data, medical history, smoking history (smokers who have smoked continuously or cumulatively for 6 months or more in their lifetime and have smoked >100 cigarettes in the previous 30 days) and/or alcohol consumption (alcohol intake ≥50 ml [50 g] per month), baseline NIHSS score, modified Rankin Scale score, Barthel score, Kolcaba's general comfort questionnaire, baseline blood pressure and blood pressure during the follow-up period, blood lipid- and blood sugar-related indicators, visual analog scale for pain scores, and treatment compliance in patients with 1-year training. Treatment compliance was assessed by calculating the proportion of patients who completed the RIC treatment, which is in line with the regularity of the trial protocol according to the RIC treatment back-end data provided by the device software that collects data via a communication chip, with ≥80% indicating good compliance and <80% indicating poor compliance.

### Statistical Analysis

Measurement data were expressed as mean ± standard deviation. An independent *t*-test was used to compare independent variables after testing for normal distribution, while Mann-Whitney *U*-test was used for non-normally distributed variables. Count data were described as frequency and percentage, and the chi-square test was used to compare the differences between the two groups. The status of patient compliance and the associated influencing factors were recorded and summarized. After all factors were compared between the groups, factors with *p*-values <0.05 were included in the multivariate logistic analysis. Multivariate logistic regression analysis (forward) was performed to detect the factors that influence compliance. All statistical analyses were performed using SPSS software, version 20.0 (SPSS, Inc., Chicago, IL, USA), and a *p*-value of <0.05 indicated significance.

## Results

### Baseline and Demographic Characteristics

**Figure 1:**
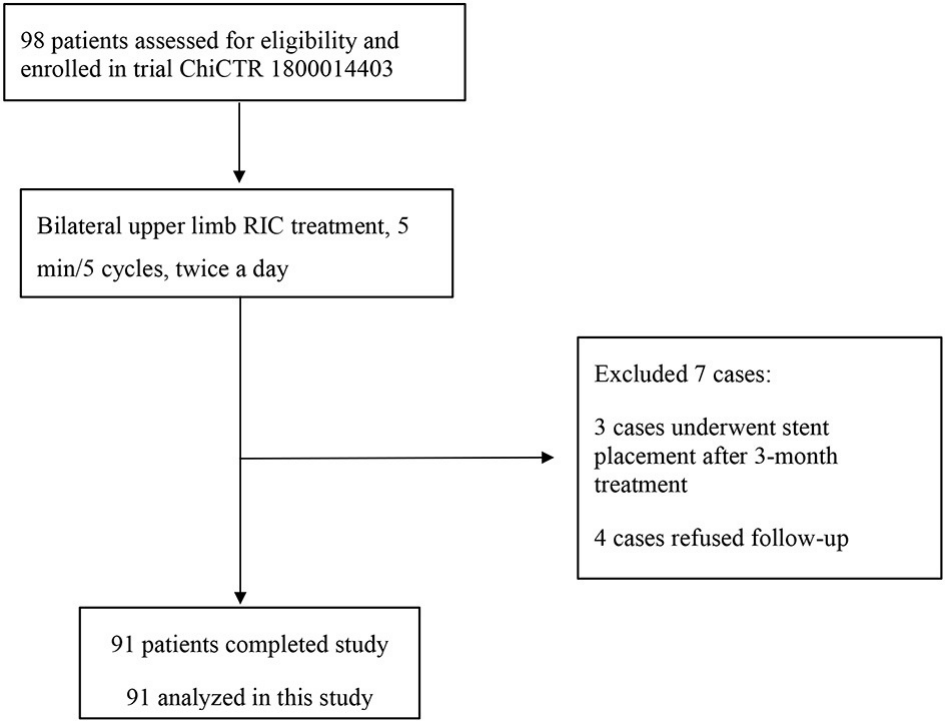
Enrollment flowchart.

A total of 91 patients were included in the final analysis (Figure 1). Their baseline and demographic data are summarized in Table 1. The mean (±SD) age was 57.98 ± 10.76 years, and 78 (85.7%) patients were male. The Han people were the most predominant ethnic group, accounting for 91.2% (*n* = 83) of all patients. The educational level of the patients was predominantly junior high school (35.16%) and senior high school (21.98%). Patients whose income was <1,000 RMB accounted for 6.6%, 1,000–3,000 RMB accounted for 22.0%, 3,000–5,000 RMB accounted for 27.5%, 5,000–10,000 RMB accounted for 30.8%, and ≥10,000 RMB accounted for 13.2% of the patients.

**Table 1:** Comparison of baseline data between the two groups.

**Item**	**Poor compliance** ***n* = 35**	**Good compliance** ***n* = 56**	**Statistics**	***p-*value**
Age	59.4 ± 9.4	57.2 ± 11.6	−0.892	0.375^a^
Male	30 (85.7)	48 (88.9)	0.013	0.909^b^
Ethnic			4.078	0.253^b^
Han	30 (85.7)	53 (94.6)		
Other	5 (14.3)	3 (5.4)		
Education			5.024	0.413^b^
Primary, below	1 (2.9)	7 (12.5)		
Junior high	15 (42.9)	17 (30.4)		
Senior high	9 (25.7)	11 (19.6)		
Vocational	2 (5.7)	2 (3.6)		
College	6 (17.1)	12 (21.4)		
Bachelor, above	2 (5.7)	7 (12.5)		
Marital status			1.939	0.379^b^
Unmarried	0 (0)	2 (3.6)		
Married	35 (100)	53 (94.6)		
Divorced	0 (0)	1 (1.8)		
Avg. monthly income			1.329	0.856^b^
≤ 1,000 RMB	3 (8.6)	3 (5.4)		
1,000–3,000	9 (25.7)	11 (19.6)		
3,000–5,000	10 (28.6)	15 (26.8)		
5,000–10,000	9 (25.7)	19 (33.9)		
≥10,000	4 (11.4)	8 (14.3)		
Residence area			1.056	0.304^b^
Rural	4 (11.4)	11 (19.6)		
Urban	31 (88.6)	45 (80.4)		
Payment method			1.213	0.750^b^
Self-pay	3 (8.6)	6 (10.7)		
Public pay	1 (2.9)	4 (7.1)		
Medical insurance	27 (77.1)	38 (67.9)		
Rural medical care	4 (11.4)	8 (14.3)		
Religious			1.115	1.000^b^
Yes	1 (2.9)	0 (0)		
No	34 (97.1)	56 (100)		
Medical history				
Hypertension	28 (80)	30 (53.6)	6.509	0.011^b^
Diabetes	15 (44.1)	18 (33.3)	1.035	0.309^b^
Hyperlipidemia	11 (31.4)	19 (33.9)	0.061	0.805^b^
Atrial fibrillation	0 (0)	2 (3.6)	1.278	0.258^b^
Stroke	8 (22.9)	11 (19.6)	0.135	0.714^b^
Heart disease	6 (17.1)	7 (12.5)	0.379	0.538^b^
Others	4 (11.4)	4 (7.4)	0.420	0.517^b^
Smoking history	16 (45.7)	31 (55.4)	1.088	0.581^b^
Alcohol consumption	16 (47.1)	25 (44.6)	0.050	0.823^b^
BMI	25.8 ± 3.2	26.2 ± 3.8	−0.526	0.600^a^
Baseline NIHSS	2 (0,4.0)	1 (0,3.0)	−1.664	0.096^c^
Baseline mRS	1 (0,3.0)	1 (0,2.0)	−1.554	0.120^c^
Baseline ADL	85.0 (70.0,95.0)	90.0 (70.0,95.0)	−0.610	0.542^c^
Baseline comfort	82.0(69.0,94.0)	91.0(78.0,98.0)	−1.372	0.170^c^
Baseline pain	0 (0, 0)	0 (0, 0)	−0.922	0.356^c^
3-month pain	0 (0, 0)	0 (0, 0)	−1.139	0.255^c^
6-month pain	0 (0, 0)	0 (0, 0)	−1.025	0.306^c^
1-year pain	0 (0, 0)	0 (0, 0)	−1.147	0.246^c^
Baseline glycosylated hemoglobin	6.2 (5.6,7.4)	5.8 (5.4,6.7)	−1.898	0.058^c^
3-month glycosylated hemoglobin	7.5 (6.3,8.1)	6.1 (5.7,7.2)	−1.628	0.104^c^
6-month glycosylated hemoglobin	5.5 (3.8,6.1)	6.2 (5.0,7.2)	−0.671	0.502^c^
1-year glycosylated hemoglobin	7.7 (5.3,8.9)	5.7 (5.4, 6.9)	−1.223	0.221^c^
Times of follow-ups	1.5 (1, 2)	2 (2, 2.75)	−2.782	0.005^c^

a *Independent t-test*;

b *Chi-square test*;

c *Mann-Whitney U-test; BMI, Body Mass Index; NIHSS, The National Institutes of Health Stroke Scale; mRS, Modified Rankin Scale; ADL, the Barthel index of activities of daily living*.

Most patients (83.52%) lived in urban areas. The method of medical payment was mainly medical insurance (71.43%). Most participants had chronic diseases, such as hypertension (63.74%), diabetes (36.26%), and hyperlipidemia (32.97%). Approximately 51.6% of the patients had a history of smoking, and 45.1% had a history of alcohol consumption. Patients with severe disease before enrollment into the study had poorer treatment compliance. Until 6 months after enrollment, patients with good compliance during the follow-up period had better blood sugar control. Comparisons of other baseline characteristics are shown in Table 1. No significant differences in all baseline characteristics were found between the two study groups (*p* < 0.05), except for the history of hypertension and number of follow-ups. Significant differences in compliance were noted (χ^2^ = 91.00, *p* < 0.001).

### Total Score of Comfort and Scores in Each Dimension

The baseline comfort of the two groups of patients undergoing RIC treatment were compared, and the results showed that the baseline Kolcaba comfort scale of patients with good compliance scores were higher than those of patients with poor compliance, physiological dimensions, 17.0 (13.0,19.0) vs 15.0 (12.0,17.0); psychological dimensions, 31.0 (27.0,35.0) vs 30.0 (25.0,34.0); sociological dimensions, 21.0 (18.0,23.0) vs 20.0 (18.0,24.0); environmental dimensions, 20.0 (17.0,22.0) vs 19.0 (12.0,24.0); and total points 91.0 (78.0,98.0) vs 82.0 (69.0,94.0) (see Table 2). Differences between groups were significant (*p* < 0.05), except for the sociological dimensions.

**Table 2:** Comparison of the Kolcaba comfort scores in each dimension of the two groups.

**Item**	**Poor compliance** ***n* = 35**	**Good compliance** ***n* = 56**	***Z***	***p-*value**
Physiological	15.0 (12.0,17.0)	17.0 (13.0,19.0)	−2.691	0.007
Psychological	30.0 (25.0,34.0)	31.0 (27.0,35.0)	−2.082	0.037
Sociological	20.0 (18.0,24.0)	21.0 (18.0,23.0)	−1.610	0.107
Environmental	19.0 (12.0,24.0)	20.0 (17.0,22.0)	−2.295	0.022
Total Score	82.0 (69.0,94.0)	91.0 (78.0,98.0)	−2.604	0.009

*The results are expressed as median (inter-quantile range). Mann-Whitney U-test was used to determine the differences between groups*.

### Factors Influencing RIC Treatment Compliance in Stroke Patients

Multivariate logistic regression analysis was performed to investigate the demographic sociological factors of the patients, pretreatment disease status of the patients, number of follow-ups, and influence of treatment comfort on patient compliance. The variables introduced into the equation were history of hypertension, number of follow-ups, and four dimensions of the comfort scale. The results show that the number of follow-ups [Adjusted OR = 2.498, 95% confidence interval (CI) 1.257–4.964] and physiological discomfort (Adjusted OR = 1.128, 95% CI 1.029–1.236) caused by RIC treatment are independent factors that influence patient compliance (Table 3).

**Table 3:** Multivariate logistic regression analysis of patient compliance.

**Item**	**B**	**SE**	***Adjusted P-*value**	**Adjusted OR**	**95% CI**
Constant	−1.961	0.781	0.012	0.141	
No. of follow-ups	0.916	0.350	0.009	2.498	1.257–4.964
Physiological comfort	0.120	0.047	0.010	1.128	1.029–1.236

*B, partial regression coefficient; SE, Standard Error; CI, confidence interval; OR, odds ratio*.

*The variables introduced into the equation were history of hypertension, number of follow-ups, and four dimensions of the comfort scale*.

*The model was adjusted for age, sex, ethnicity, education, marital status, average monthly income, residence area, health payment method, religion, smoking history, and drinking history*.

## Discussion

In this study, we found that in patients with ischemic stroke who received prolonged RIC treatment, the number of follow-ups during the study period was associated with patients' compliance to RIC, and the physiological discomfort related to RIC appears to decrease patients' compliance.

The results of this study showed that the number of follow-ups was positively correlated with patient compliance, and the number of follow-ups was an independent influencing factor of patient compliance. Several studies (10–13) have shown that patients' fear of the potential or actual adverse consequences or disappearance of symptoms can decrease patients' compliance. An effective way of solving the above problems is to communicate with patients to resolve their concerns and convey the rationale and importance of treatment; to elicit and solve specific problems, the above methods can be used to improve treatment compliance. In a long-term treatment study, this can be achieved by increasing the number of follow-ups and by regularly communicating with patients to solve their practical problems.

In this study, the number of follow-up visits was one of the independent factors that influenced patients' compliance to RIC therapy. Therefore, the number of follow-ups should be increased for patients receiving long-term RIC treatment, and at each visit, it is important to make enquiries regarding the patients' doubts and questions, to establish a good relationship with patients and their families, and to provide systematic and continuous health education to correct the patients' wrong attitudes and beliefs. Furthermore, with the rapid development of mobile devices and applications in the field of medicine, appropriate use of these novel techniques may increase communication with patients and this could improve their compliance.

The results of this study indicate that physiological discomfort is an independent influencing factor that reduces patient compliance. Comfort is a subjective sensation that comprises both physical and psychological dimensions (14). RIC treatment uses pressure to block blood flow, and this causes pain and numbness, which in turn leads to physiological discomfort. The duration of such discomfort affects the patient's psychological well-being, and this may influence patients' compliance to the RIC treatment, which not only affects the therapeutic effects of RIC but may also lead to drop-out in clinical research (15). Previous studies have shown that complexity, comfort, and duration of treatment programs influence patient compliance (16). Furthermore, the level of comfort had an increasing trend along with continuation of treatment, which indicates that as the test progressed over time, participants gradually adapted to the discomfort at the strongest pressure per cycle, although the discomfort still existed. Therefore, a more comfortable and equally effective RIC treatment protocol can improve the compliance of patients to RIC treatment.

Currently, numerous research teams are actively engaged in clinical transformation research because of the importance of RIC and its broad clinical application prospects (15, 17). However, the clinical transformation of RIC is still in its initial stages. To ensure that the clinical transformation of RIC achieves better results, an evidence-based supportive nursing process is necessary. Therefore, in future studies, researchers should comprehensively consider the patient's efficacy, comfort, compliance, and other factors, while choosing the optimal treatment dose, and improve the RIC nursing process accordingly, which will provide the basis for clinical implementation and scientific research of RIC.

This study has several limitations. First, this was a retrospective study that was based on a randomized controlled trial. This study design has an inherent limitation as we could only use the existing data for analysis. In the future, a prospective research will be necessary to evaluate more factors that may affect patient compliance to RIC treatment. Second, Kolcaba comfort scale is a subjective evaluation tool that may be limited by self-report biases; thus, the findings of this study should be interpreted with caution. In addition, the sample size was small, and some known and unknown factors may have influenced the results. Therefore, larger studies are needed to confirm these results. Finally, our study only focused on patients' factors, and whether their family and social support influenced compliance to RIC treatment needs further investigation.

In conclusion, in patients with ischemic cerebrovascular disease who were treated with RIC, the number of follow-up visits and the physiological discomfort associated with RIC were independent influencing factors of patient compliance. Further studies are needed to confirm these results and determine appropriate RIC protocols and their corresponding nursing models.

## Data Availability Statement

The raw data supporting the conclusions of this article will be made available by the authors, without undue reservation.

## Ethics Statement

The study protocol was approved by the Ethics Committee of Xuanwu Hospital, Capital Medical University (2018009). The patients/participants provided their written informed consent to participate in this study.

## Author Contributions

JZ and HC: study concept and design, critical revision of the manuscript for important intellectual content, and study supervision. JZ: acquisition, analysis and interpretation of data, and drafting of the manuscript. KF: analysis and interpretation of data. WZ: revision of the manuscript. HY and JM: acquisition of data. All authors read and approved the final manuscript.

## Conflict of Interest

The authors declare that the research was conducted in the absence of any commercial or financial relationships that could be construed as a potential conflict of interest.

## Publisher's Note

All claims expressed in this article are solely those of the authors and do not necessarily represent those of their affiliated organizations, or those of the publisher, the editors and the reviewers. Any product that may be evaluated in this article, or claim that may be made by its manufacturer, is not guaranteed or endorsed by the publisher.
